#  Evaluation of Axillary Lymph Node Marking with Magseed® before and after Neoadjuvant Systemic Therapy in Breast Cancer Patients: MAGNET Study

**DOI:** 10.1155/2022/6111907

**Published:** 2022-07-09

**Authors:** María Martínez, Sara Jiménez, Florentina Guzmán, Marta Fernández, Elena Arizaga, Consuelo Sanz

**Affiliations:** ^1^Radiology Department, Hospital General Universitario Morales Meseguer, Avda. Marqúes de Los Vélez S/n, Murcia 30007, Spain; ^2^Radiology Department, Hospital Universitario 12 de Octubre, Av. de Córdoba S/n, Madrid 28041, Spain; ^3^Radiology Department, Hospital Virgen de La Arrixaca, Ctra. Madrid-Cartagena S/n, Murcia 30120, Spain; ^4^Gynaecology Department, Hospital Universitario Donostia, Begiristain Doktorea Pasealekua, S/n, Donostia 20014, Gipuzkoa, Spain; ^5^Radiology Department, Hospital Universitario Donostia, Begiristain Doktorea Pasealekua, S/n, Donostia 20014, Gipuzkoa, Spain; ^6^Gynaecology Department, Hospital Universitario 12 de Octubre, Av. de Córdoba, S/n, Madrid 28041, Spain

## Abstract

**Background:**

Due to the high false negative rate (FNR) associated with sentinel lymph node biopsy (SLNB) after neoadjuvant systemic therapy (NAST), the standard surgical treatment for patients with an initially positive axilla and indicated for NAST is axillary lymph node dissection (ALND). To avoid unnecessary ALND, this multicenter, prospective, observational study aimed to determine the effectiveness and ease of using magnetic seeds (Magseed®) for targeted axillary dissection (TAD) when the seeds are placed before or after NAST.

**Materials and Methods:**

We recruited 81 patients diagnosed with T1-T3 breast cancer, with clinically/radiologically positive nodal involvement (cN1, 75 patients with 1–3 nodes suspected nodes and 6 patients with up to 4 suspected nodes) prior to NAST. Positive nodes detected by fine-needle aspiration biopsy or core needle biopsy were marked with a stainless steel marker coil and after NAST with Magseed® prior to surgery (Post-NAST group), or directly with Magseed® before NAST (Pre-NAST group). The correlation between lymph nodes marked with Magseed® (MLNs) and sentinel lymph nodes (SLNs) was calculated based on pathologic assessment with the OSNA assay (Sysmex Corporation, Kobe) or conventional sectioning and staining techniques according to the standard protocols of each center.

**Results:**

All magnetic seeds were successfully identified and retrieved in just over 10 minutes of surgery, guided by the Sentimag® magnetometer system. The overall concordance rate between MLNs and SLNs was 81.5%, and the concordance between MLNs and SLNs with metastasis was 93.8%. Metastasis was detected in 54.3% of the MLNs compared with 48.1% of SLNs. In cases that presented negative MLN and negative SLN (negative TAD), the FNR was 0%. No significant differences were found between the Post-NAST and Pre-NAST groups.

**Conclusions:**

Our results validate the use of Magseed® for long-term marking of axillary lymph nodes and show that when used in combination with SLNB for TAD, a FNR of 0% can be achieved, avoiding unnecessary ALND.

## 1. Introduction

The most important prognostic factor in patients diagnosed with breast cancer is the presence or absence of regional metastasis in axillary lymph nodes [1]. The removal of most or all the axillary lymph nodes, axillary lymph node dissection (ALND), has been the standard technique used in the staging and treatment of breast cancer patients with lymph node involvement. However, it is increasingly associated with complications such as lymphedema and neuropathic pain [2].

Sentinel lymph node biopsy (SLNB), which involves finding and removing the first lymph node(s) to which a tumor is likely to spread, was developed as an alternative, less invasive approach for assessing axillary lymph node status. In early-stage breast cancer patients (clinically node negative, cN0), a negative or low metastatic volume result of SLNB can be used to avoid ALND [3–5]. Among women with cT1-2N0 breast cancer and metastases to 1-2 SLNs undergoing breast-conserving surgery, whole-breast irradiation, and adjuvant systemic therapy, the 10-year overall survival of those treated with SLNB alone was noninferior to those who underwent ALND [6]. However, it is still not clear whether ALND can be omitted in the case of SLN involvement after NAST.

Neoadjuvant systemic therapy (NAST) is increasingly used in the treatment of breast cancer to reduce tumor bulk and the extent of surgery. It also offers the opportunity to measure the response to systemic therapy in both the primary tumor and axilla. In fact, a pathologic complete response (pCR) in axillary lymph nodes after NAST can be considered an early surrogate marker of the long-term outcome [7].

In most cases, the standard surgical treatment for patients with an initially positive axilla and indicated for NAST is ALND, because NAST can increase the false negative rate (FNR) of SLNB [8–10]. Further research into the feasibility of Post-NAST SLNB in pathologically proven node-positive cases before NAST is required to avoid unnecessary ALND in patients that show a pathologic complete axillary response [11].

Surgical removal of marked positive lymph nodes, using wires, biopsy markers (coils, clips), radioactive seeds, or magnetic seeds, in combination with SLNB after NAST, known as targeted axillary dissection (TAD), can reduce the FNR of SLNB [12–15].

Tumor-positive axillary lymph nodes can be marked preoperatively with radioactive seeds. These can be placed several days before surgery and recovered using a gamma probe but can only be carried out in hospitals with nuclear medicine facilities [12, 16]. This limitation can be overcome using magnetic seeds approved for long-term use, which can be placed before NAST and before surgery and are easily recovered without nuclear medicine services. Several studies have shown that the inducible magnetic seed, Magseed® (Endomag, UK), can be used to localize axillary lymph nodes preoperatively safely and accurately [17, 18].

Most studies to date have examined the use of Magseed® placement after NAST [19]. The aim of this one-year, multicenter study was to assess the reliability of lymph node marking with Magseed® both before and after NAST, as well as seed retrieval during surgery with a handheld magnetometer system (Sentimag®), in patients diagnosed with T1-T3 breast cancer involvement (cN1, 1–4 suspected nodes) prior to NAST. Our results contribute to validate the use of Magseed® for long-term marking and highlight the potential of using Magseed® to de-escalate surgical management of the axilla, which could contribute to improve patients' quality of life without compromising cancer-treatment outcomes.

## 2. Materials and Methods

### 2.1. Study Design and Subjects

MAGNET was a prospective, observational study, involving 81 adult patients between December 2018 and December 2019 across four university hospitals in Spain.

Eighty-one patients diagnosed with T1-T3 breast cancer, with clinically/radiologically positive nodal involvement (cN1, 1–4 suspected nodes) and indicated for NAST, were eligible for the study. Fine-needle aspiration (FNA) biopsy or core needle biopsy of the most morphologically suspect and caudal node was performed to detect cancer cells. Positive nodes detected by fine-needle aspiration biopsy were marked with a stainless steel marker coil (Post-NAST group) or Magseed® (Pre-NAST group). If there were two equally suspicious nodes, the most cranial and the most caudal were analysed and, if positive, marked.

Patients with T4 tumors; supra/infraclavicular lymph node involvement or internal mammary chain (cN3) and presence of palpable lymph node conglomerate at diagnosis; previous ipsilateral breast or axillary surgery or absence of axillary surgery after NAST; or distant metastasis at diagnosis were excluded from the study.

The primary endpoint was to determine the safety and reliability of Magseed® marking of positive lymph nodes before and after NAST and subsequent retrieval during surgery.

**Figure 1:**
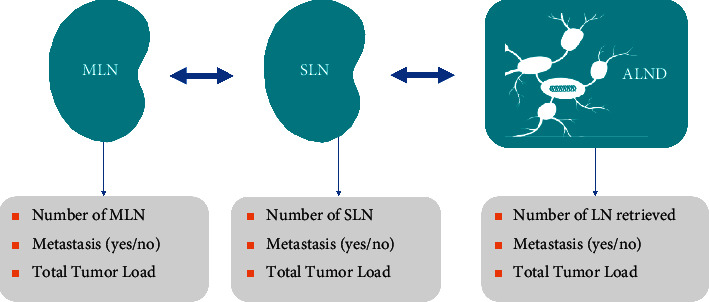
Technical variables of the MLN, SLN, and ALND.

Secondary endpoints were as follows: ultrasound identification rate of the magnetic nodal seeds after NAST, surgery time required to retrieve them, the number of marked lymph nodes (MLNs) obtained in surgery, the number of MLNs retrieved in the pathology lab, the number of sentinel lymph nodes (SLNs) obtained in surgery, the number of SLNs retrieved in the pathology lab, the correlation between MLNs and SLNs, the number of nodes obtained in ALND (in patients who underwent ALND), tumor load (if there were metastases), and TAD FNR (see Figure 1 for variables of interest).

The study was approved by the ethical committee of the participating centers and was conducted in accordance with Good Clinical Practice guidelines and the Declaration of Helsinki of the World Medical Association. All patients gave their written informed consent for inclusion in the study.

Data were recorded in an electronic case report form and anonymized. All data were considered confidential and treated in accordance with the Spanish Law on the Protection of Personal Data.

### 2.2. Method

**Figure 2:**
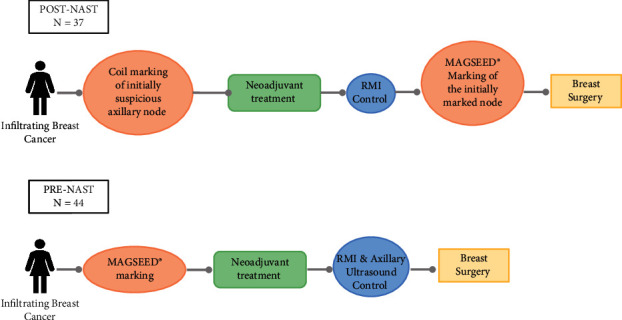
Patient flowchart. All lymph nodes marked with Magseed® were identified by axillary ultrasound, and all marked lymph nodes were removed during surgery.

An axillary ultrasound reviewing Berg levels I, II, and III was performed on all participating patients (see Figure 2). In patients with suspected nodes at level I (UN4-suspicious (a cortex with uniform cortical thickness ≥2.3 mm) and UN5-replaced (an enlarged node with no fatty hilum) according to Amonkar's nodal scoring classification based on morphological features on ultrasound [20]), FNA or core needle biopsy of the most caudal and morphologically suspect node was performed. If positive for malignancy, the node was marked with a stainless steel marker coil and after NAST, with Magseed® up to 30 days prior to surgery (Post-NAST group), or, after EC approval for use in soft tissue with no restrictions on the length of time that the marker can remain in the body (24^th^ February 2020), with Magseed® from the beginning, before starting NAST (Pre-NAST group). After a similar number of patients were in the Post- and Pre-NAST group, patients were marked consecutively.

A single seed was placed in all but one case, in which two seeds were placed, one in the most caudal and one in the most cranial node that looked equally suspicious. The magnetic seeds were guided by ultrasound and released in the cortical area of maximum thickness as previously described [14]. The release of the marker was confirmed by ultrasound and mammogram.

SLNB was carried out according to the protocol of each center.

On the day of surgery, the node marked with Magseed® was recovered, guided by the Sentimag® magnetometer system (Endomag, UK).

Both MLNs and SLNs were submitted for pathologic assessment with the OSNA assay (Sysmex Corporation, Kobe) [21] or conventional sectioning and staining techniques according to the standard protocols of each hospital.

To validate the results of TAD, ALND was carried out in 75 patients according to each hospital's protocol and non-MLN/non-SLN lymph nodes were assessed for metastasis. ALND was not performed in 6 patients who had negative SLNs and negative MLNs.

### 2.3. Data Analyses

For the statistical analysis of the data, positive nodes were defined by the presence of isolated tumor cells, macrometastases, and micrometastases. False negative MLNs were defined as those without metastases when other ALND nodes were positive including SLN.

For the descriptive analyses, data were separated into two groups (Pre-NAST and Post-NAST). The results are given for every group and the overall population. For quantitative variables, the following statistics were computed: N, mean, SD, 95% CI, median, P25 and P75, minimum, and maximum. For qualitative variables, the following statistics were computed: N and frequencies for every category with respective percentages. The same analyses were carried out in the Post-NAST group and Pre-NAST group separately.

The Mann–Whitney test and Fisher's exact test were used to compare the results in the Pre- and Post-NAST groups (Supplementary Table 1). The results of the observational study of cohorts are descriptive and exploratory, and further research would be needed to confirm the results.

## 3. Results

### 3.1. Patient Characteristics

Eighty-one patients were included in the study. In the Post-NAST group (37 patients; 45.7%), positive lymph nodes were marked with a stainless steel marker coil before NAST and with Magseed® after NAST and before surgery. The Pre-NAST group (44 patients; 54.3%) had their lymph node(s) marked with Magseed® before they received NAST (Pre-NAST group) (Figure 2).

The median age of patients was 47 years (range: 29–78 years old), and the average initial tumor size was 4 cm (±2.9) (Table 1). 81 patients (100%) were classified as having cN1 breast cancer (1–4 suspected nodes). The molecular subtypes of breast cancer were as follows: 41 patients (50.6%) had luminal B breast cancer, 27 (33.3%) had HER2+/EP + breast cancer, 10 (12.3%) had TN breast cancer, 2 (2.5%) had luminal A breast cancer, and 1 (1.25%) luminal B/HER2^+^ breast cancer.

After the completion of NAST, an axillary ultrasound was performed to assess the clinical response. 62.9% of patients underwent breast conserving surgery (BCS), and 37% underwent mastectomy (MT). 34/79 patients (43%) achieved a pathological complete response (pCR), defined as disappearance of cancer cells both in the breast and axilla after neoadjuvant treatment.

## 4. Magseed® Marking of Axillary Nodes

### 4.1. Overall Findings

All magnetic seeds were successfully identified and retrieved in just over 10 minutes of surgery. All SLNs were detected according to the SLNB protocol of each center.

On average, in TAD, 1.5 (±1.2) nodes were retrieved per patient and the median number of nodes retrieved per patient was 1 (range: 1–8). The overall concordance rate between SLNs and MLNs was 81.5% (Table 2).

The concordance between SLNs and MLNs with metastasis was 93.8%. In the 6.2% discordant cases (*N* = 5), metastasis was only found in the MLN, the SLN being negative. Overall, metastases were detected in 54.3% of the MLNs compared with 48.1% of SLNs.

Seventy-five of the 81 patients (92.6%) underwent ALND. ALND was not carried out in 6 patients with a negative TAD, as specified in the center's protocol. A total of 638 lymph nodes were excised, with a median of 10 (range: 1–26) lymph nodes removed per patient. Metastasis in non-SLNs or non-MLNs was detected in 21 (28%) patients.

In patients who presented negative SLN and negative MLN (negative TAD), the ALND result was negative, demonstrating that the FNR in these cases is 0% (Table 3).

44 (54.32%) patients did not achieve a pathological complete response in the axilla, and in 100% of these cases, metastases were detected in the MLN.

### 4.2. Post-NAST and Pre-NAST Group Findings

We found no significant differences between the Post-NAST and Pre-NAST groups, indicating that lymph node marking with Magseed® before NAST is just as reliable as marking after NAST.

In the Post-NAST group (*N* = 37; 45.7%), the overall concordance rate between SLNs and MLNs was 86.5%.

In the Post-NAST group, metastases were detected in 45.9% of MLNs compared with 43.2% of SLNs and the concordance between these nodes was 97.3%. In the 2.7% discordant cases (1 patient), metastasis was only found in the MLN and the SLN was negative (Table 3).

31 patients (83.8%) in this group underwent ALND. A total of 268 lymph nodes were retrieved, with an average of 9.9 (±5) lymph nodes removed per patient. Metastasis in non-SLN or non-MLN was detected in 7 (22.6%) patients, all of whom had a positive SLN and a positive MLN (Table 3).

In the Pre-NAST group (*N* = 44; 54.3%), the overall concordance rate between SLNs and MLNs was 77.3%.

In the Pre-NAST group, metastases were detected in 61.4% of MLNs compared with 52.3% of SLNs and the concordance between these nodes was 90.9%. In the 9.1% discordant cases (4 patients), metastasis was only found in the MLN and the SLN was negative (Table 3).

All patients in this group (44) underwent ALND. A total of 370 lymph nodes were retrieved, with an average of 10.3 (±4.3) lymph nodes removed per patient. Metastasis in non-SLN or non-MLN was detected 14 (31.8%) patients, all of whom had a positive MLN (Table 3).

### 4.3. Safety Results

No complications due to Magseed® deployment or identification were observed.

## 5. Discussion

Our one-year, multicenter study confirms the reliability of lymph node marking with Magseed® both before and after NAST in patients diagnosed with T1-T3 breast cancer with clinically/radiologically positive nodal involvement (cN1, 1–4 suspected nodes) prior to NAST.

We found no significant differences in the overall concordance of MLNs and SLNs between patients with nodes marked using Magseed® after NAST (Post-NAST group) and before NAST (Pre-NAST). Similarly, no differences were found in the concordance of MLNs and SLNs with metastasis between the two groups. Marking positive nodes with Magseed® early (before NAST) is safe and does not interfere with MRI scanning.

In our study, in cases that presented a negative Magseed®-MLN and a negative SLN, the FNR was 0%, indicating that Magseed® is a fast, simple, and safe way to accurately detect the response of a positive lymph node to NAST.

We are confident in the 0% FNR result as the study was carried out across four hospitals with extensive experience in marking with clips, by surgery and pathology teams specializing in labeled lymph node analysis and ultrasound staging. Most patients had limited axillary metastatic involvement (1 to 3 suspected nodes, with the exception of 6 cases with 4 nodes) and thus a greater probability of complete response and a lower FNR.

Our concordance rate is similar to that reported in a study by Simons et al. [22] showing that the Sentimag®-MLN matched the SLN in 80% (40/50) of patients.

We obtained a higher concordance rate between MLNs and SLNs than Mariscal Martínez et al. [14] Their study found that in breast cancer patients with axillary lymph node involvement treated with neoadjuvant chemotherapy, the Sentimag®-MLN corresponded to the SLN in 50% of cases. They, and others [10, 23], have shown that removing SLNs and MLNs can reduce the FNR associated with SLNB alone from well over 10% to between 6 and 2%.

In our study, discordant cases (*N* = 5) were due to the MLN being positive and the SLN negative, suggesting that Magseed® could be a more sensitive method for detecting metastasis.

In most studies on lymph node marking with magnetic seeds, the seeds are placed after NAST [19]. Our results indicate that placing them earlier does not interfere with patients' follow-up, regardless of the molecular classification of their cancer and response to NAST.

Magseed® is approved for long-term use in any soft tissue [24] and offers various advantages over other marking methods, the main one being that X-ray is not required to locate the MLN after NAST. Magnetic seeds can be firmly and precisely implanted, aiding surgery, and are easily retrieved without the regulatory issues associated with radioactive seeds. In our study, all magnetic seeds in the Pre- and Post-NAST groups were successfully retrieved in a similar amount of surgery time (10 minutes) with a handheld magnetometer system (Sentimag®).

We did not investigate as the primary objective of the study whether Magseed® led to signal void artifacts during follow-up breast MRI scans, although it was internally assessed. MRI artifacts do not affect the ultrasound assessment of the axilla.

The limitations of this study include the relatively small sample size, the fact that lymph nodes after NAST were analysed using two different methods (OSNA and hematoxylin-eosin), and that ALND was not completed in all cases.

Results from the RISAS trial [25] and preliminary results from the MAGELLAN trial [26], which are examining the use of radioactive iodine seeds and magnetic seeds to mark axillary lymph nodes and guide surgical localization in patients with node-positive breast cancer following NAST, will further contribute to determining whether TAD is a valid option for assessing the axillary response to NAST and could be used to avoid unnecessary ALND.

## 6. Conclusions

According to the latest report by the National Comprehensive Cancer Network (NCCN), TAD after NAST is becoming part of the standard approach to treating patients with initial biopsy-proven node-positive breast cancer. Our results support the use of Magseed® before NAST for TAD in these patients to avoid unnecessary ALND.

## Figures and Tables

**Table 1:** Patient characteristics.

Characteristics	Overall (%)	Patients (Post-NAST) (%)	Patients (Pre-NAST) (%)
Number of patients (n)	81 (100)	37 (45.7)	44 (54.3)
Age			
Mean (SD)	50.7 (12.7)	50.9 (10.9)	50.6 (14.1)
95% CI	(48.0; 53.5)	(47.4; 54.4)	(46.4; 54.7)
Median (min; max)	47.0 (29.0; 78.0)	47.0 (33.0; 78.0)	47.0 (29.0; 74.0)
P25; P75	41.0; 61.0	44.0; 56.0	40.0; 63.8
Initial tumor size (cm)			
Mean (SD)	4.0 (2.9)	3.4 (2.1)	4.5 (3.4)
95% CI	(3.4; 4.7)	(2.8; 4.1)	(3.5; 5.6)
Median (min; max)	3.1 (0.0; 16.0)	2.8 (0.0; 9.1)	3.2 (1.5; 16.0)
P25; P75	2.3; 5.3	2.3; 3.6	2.6; 5.6
Initial lymph node staging			
cN1	81 (100)	37 (100)	44 (100)
Tumor pathological response (Post-NAST)			
Patients	79^*∗*^	36	43
pPR	45 (56.9)	18 (50.0)	27 (62.8)
pCR	34 (43)	18 (50.0)	16 (37.2)
Surgical procedure on the breast			
Patients	81	37	43
BCS	51 (62.9)	26 (70.3)	25 (58.1)
MT	30 (37)	11 (29.7)	18 (41.9)
Molecular classification			
Patients	81	37	44
HER + EP^+^	27 (33.3%)	17 (45.9%)	10 (22.7%)
LB	41 (50.7%)	16 (43.3%)	25 (56.9%)
TN	10 (12.3%)	4 (10.8%)	6 (13.6%)
LA	2 (2.5%)	0 (0%)	2 (4.5%)
LB/HER2^+^	1 (1.2%)	0 (0%)	1 (2.3%)

^*∗*^Information missing for 2 cases. BCS, breast-conserving surgery; EP, estrogen and progesterone receptor; HER2 human epidermal growth factor receptor; LA, luminal A; LB, luminal B; MT, mastectomy; NAST, neoadjuvant systemic therapy; pCR, pathological complete response; pPR, pathological partial response; TN, triple negative.

**Table 2:** Lymph node marking with Magseed®.

	Overall (%)	Patients (Post-NAST) (%)	Patients (Pre-NAST) (%)
Identification and retrieval of Magseed® marked nodes			
Patients (n)	81	37	44
No	0 (0)	0 (0)	0 (0)
Yes	81 (100.0)	37 (100.0)	44 (100.0)
Concordance between SLN and MLN			
No	15 (18.5)	5 (13.5)	10 (22.7)
Yes	66 (81.5)	32 (86.5)	34 (77.3)
Nodes retrieved per patient in TAD			
Mean (SD)	1.5 (1.2)	1.5 (1.1)	1.5 (1.3)
95% CI	(1.3; 1.8)	(1.2; 1.9)	(1.2; 1.9)
Median (min; max)	1.0 (1.0; 8.0)	1.0 (1.0; 6.0)	1.0 (1.0; 8.0)
P25; P75	1.0; 1.0	1.0; 2.0	1.0; 1.0
Total SLNs retrieved (n)	200	98	102
Total MLNs retrieved (n)	98	45	53
Metastasis in MLN			
No	37 (45.7)	20 (54.1)	17 (38.6)
Yes	44 (54.3)	17 (45.9)	27 (61.4)
Metastasis in SLN			
No	42 (51.9)	21 (56.8)	21 (47.7)
Yes	39 (48.1)	16 (43.2)	23 (52.3)
Metastasis in ALND (in non-SLNs or non-MLNs)			
Patients (n)	75	31	44
No	54 (72)	24 (77.4)	30 (68.2)
Yes	21 (28)	7 (22.6)	14 (31.8)
Nodes retrieved per patient in ALND			
Mean (SD)	10.1 (4.6)	9.9 (5.0)	10.3 (4.3)
95% CI	(9.0; 11.3)	(8.0; 11.8)	(8.9; 11.7)
Median (min; max)	10.0 (1.0; 26.0)	10.0 (1.0; 26.0)	10.0 (2.0; 24.0)
P25; P75	8.0; 12.0	6.5; 12.0	8.0; 11.0
Total nodes retrieved in ALND (n)	638	268	370

ALND, axillary lymph node dissection; MLN, marked lymph node; NAST, neoadjuvant systemic therapy; SLN, sentinel lymph node; SLNB, sentinel lymph node biopsy; TAD, targeted axillary dissection.

**Table 3:** Comparison between metastases in MLN and/or SLN and ALND result.

Group	SLN	MLN	Number of patients	ALND^+^	%
Pre-NAST	+	+	23	13	56.5
	+	−	0	0	0.0
	−	+	4	1	25.0
	−	−	17	0	0.0

Post-NAST	+	+	16	7	43.8
	+	−	0	0	0.0
	−	+	1	0	0.0
	−	−	14	0	0.0

## Data Availability

The data presented in this study are not openly available due to conﬁdentiality reasons but are available upon reasonable request to the corresponding authors.

## Supplementary Materials

Supplementary Materials Supplementary Table 1: comparison between Pre- and Post-NAST groups.
