# Supplementary material for: Evaluation of Axillary Lymph Node Marking with Magseed® before and after Neoadjuvant Systemic Therapy in Breast Cancer Patients: MAGNET Study
Source: Breast J. 2022 Jul 9;2022:6111907. doi: 10.1155/2022/6111907 (PMC9288346; doi:10.1155/2022/6111907)
Supplement: Supplementary Materials — Supplementary Table 1: comparison between Pre- and Post-NAST groups. [file 6111907.f1.docx]

| Supplementary Table 1: Comparison between the Pre- and Post- NAST groups* | | |
| --- | --- | --- |
|  | p-value | test |
| Number of marked lymph nodes recovered | 0.7114 | Mann-Whitney test |
| Surgical time for recovery of the marked node (minutes) | 0.6361 | Mann-Whitney test |
| Metastasis in marked lymph node | 0.1858 | Fisher's exact test |
| TTL in labeled node | 0.2345 | Mann-Whitney test |
| Concordance between sentinel node and marked node | 0.3919 | Fisher's exact test |
| Total number of lymph nodes obtained after lymphadenectomy | 0.9721 | Mann-Whitney test |
| Metastasis in lymphadenectomy | 0.4369 | Fisher's exact test |

* p-values for the parameters compared to determine the reliability of Magseed® in both groups.
